# Correction to: Cell wall components and pectin esterification levels as markers of proliferation and differentiation events during pollen development and pollen embryogenesis in *Capsicum annuum* L.

**DOI:** 10.1093/jxb/eraf162

**Published:** 2025-07-03

This is a correction to: Ivett Bárány, Begoña Fadón, María C. Risueño, Pilar S. Testillano, Cell wall components and pectin esterification levels as markers of proliferation and differentiation events during pollen development and pollen embryogenesis in *Capsicum annuum* L., *Journal of Experimental Botany*, Volume 61, Issue 4, March 2010, Pages 1159–1175, https://doi.org/10.1093/jxb/erp392

In June 2024, a reader informed the journal about concerns regarding some figures in this article. Following an investigation by the journal, conducted in accordance with guidelines established by the Committee on Publication Ethics, the authors would like to correct some mistakes in their article.

The authors used an incorrect image for Figure 6D during figure preparation. The authors have replaced this image with the correct image in the revised Figure 6 below.

Figure 7H’ shows repeated image sections that are not present in the underlying original image that was provided by the authors. The origin of the repetitions in Figure 7H’ could not be established. The authors have replaced this image with the correct image in the revised Figure 7 below.

These mistakes do not compromise the conclusions of the study. The authors sincerely apologize for any inconvenience caused by these oversights. The figures have been corrected only in this correction notice to preserve the published version of record.


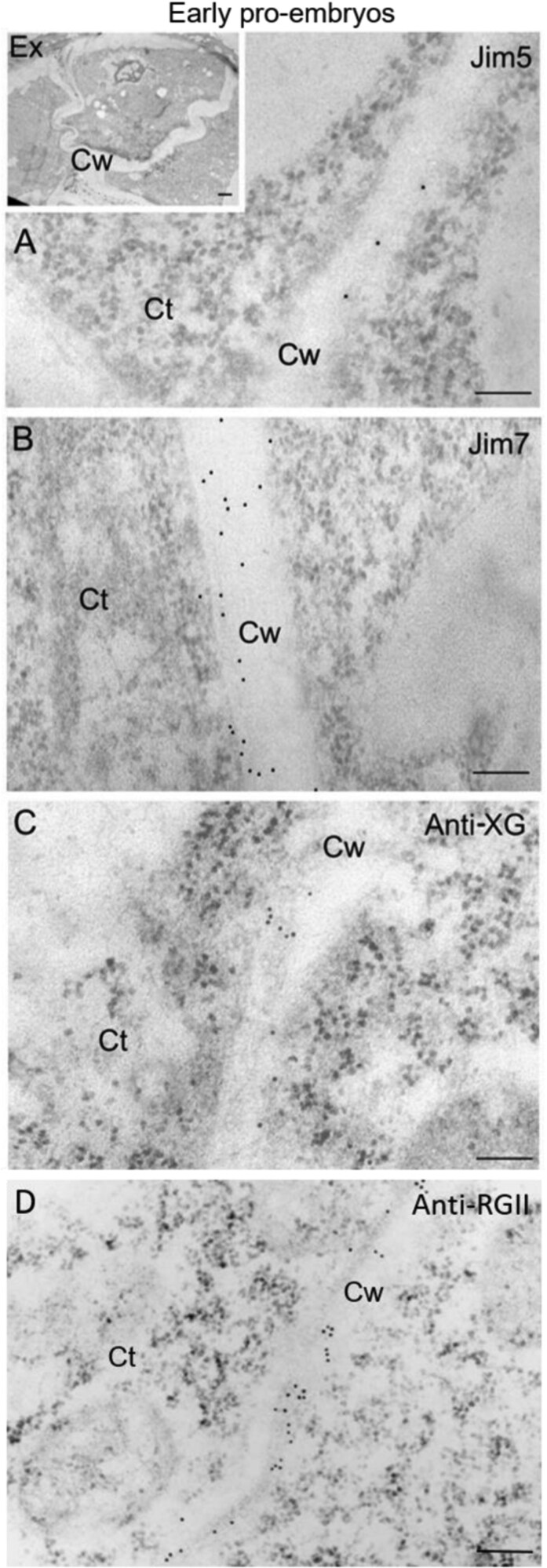


Fig. 6. Immunogold labelling of cell wall antigens in early pollen-derived proembryos of *Capsicum annuum* L. Electron micrographs of Lowicryl ultra-thin sections. Insert: low magnification micrograph showing proliferating cells of an early proembryo still surrounded by the special pollen wall, the exine (Ex). (A, B, C, D) High magnification micrographs showing the immunogold labelling with JIM5 (A), JIM7 (B), anti-XG (C), and anti-RGII (D) in the cell walls (Cw). Ct, cytoplasm. Bars: (A, B, C, D) 200 nm; insert: 1 μm.


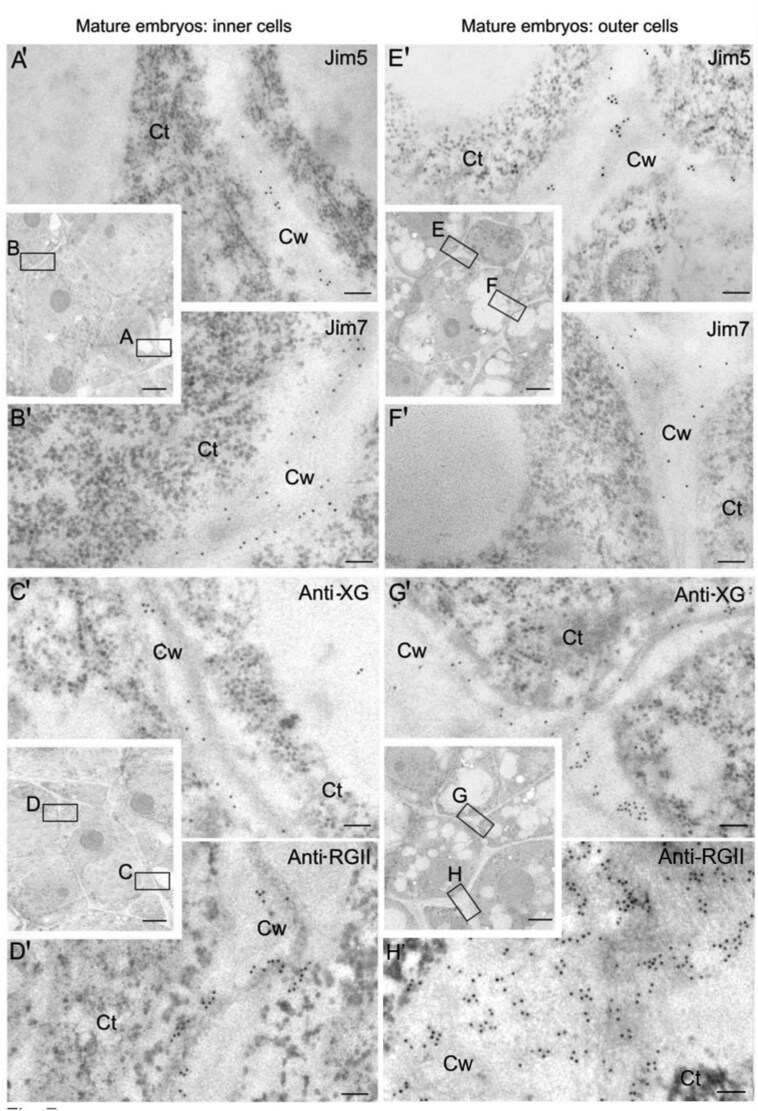


Fig. 7. Immunogold labelling of cell wall antigens in the two cell types of late-mature pollen-derived embryos of *Capsicum annuum* L. Electron micrographs of Lowicryl ultrathin sections. Inserts: low magnification micrographs showing the structural organization of the cells of the inner and outer areas of the mature embryo. Location and distribution at the ultrastructural level of JIM5, JIM7, anti-XG, and anti-RGII antigens in the newly-formed walls (squares A, B, C, D) of the inner proliferating cells (A′, B′, C′, D′) and in the more developed walls (squares E, F, G, H) of the outer differentiating cells (E′, F′, G′, H′) of the mature embryo. Ct, cytoplasm; Cw, cell wall. Bars: (A′-H′) 200 nm; inserts: 1 μm.

